# The Influence of Clusterin Glycosylation Variability on Selected Pathophysiological Processes in the Human Body

**DOI:** 10.1155/2022/7657876

**Published:** 2022-08-28

**Authors:** Ewa Janiszewska, Agnieszka Kmieciak, Monika Kacperczyk, Aleksandra Witkowska, Ewa Maria Kratz

**Affiliations:** Department of Laboratory Diagnostics, Division of Laboratory Diagnostics, Faculty of Pharmacy, Wroclaw Medical University, Borowska Street 211A, 50-556 Wroclaw, Poland

## Abstract

The present review gathers together the most important information about variability in clusterin molecular structure, its profile, and the degree of glycosylation occurring in human tissues and body fluids in the context of the utility of these characteristics as potential diagnostic biomarkers of selected pathophysiological conditions. The carbohydrate part of clusterin plays a crucial role in many biological processes such as endocytosis and apoptosis. Many pathologies associated with neurodegeneration, carcinogenesis, metabolic diseases, and civilizational diseases (e.g., cardiovascular incidents and male infertility) have been described as causes of homeostasis disturbance, in which the glycan part of clusterin plays a very important role. The results of the discussed studies suggest that glycoproteomic analysis of clusterin may help differentiate the severity of hippocampal atrophy, detect the causes of infertility with an immune background, and monitor the development of cancer. Understanding the mechanism of clusterin (CLU) action and its binding epitopes may enable to indicate new therapeutic goals. The carbohydrate part of clusterin is considered necessary to maintain its proper molecular conformation, structural stability, and proper systemic and/or local biological activity. Taking into account the wide spectrum of CLU action and its participation in many processes in the human body, further studies on clusterin glycosylation variability are needed to better understand the molecular mechanisms of many pathophysiological conditions. They can also provide the opportunity to find new biomarkers and enrich the panel of diagnostic parameters for diseases that still pose a challenge for modern medicine.

## 1. Introduction

Each year, medical research provides a large amount of significant information that contributes to improved disease diagnosis as well as treatment development. Although the achievements of medicine provide hope and the chance for a healthier, longer life for many patients, numerous cases still pose a challenge to clinicians and researchers. One of the most important and popular directions of scientific research in medicine is based on the recognition of human disease mechanisms, including those occurring on a molecular level, the understanding of which forms the basis for the development of new personalized therapies and, as a consequence, typing more sensitive and specific biomarkers to enrich medical laboratory diagnostics. The primary aim of many research projects is to propose effective diagnostic parameters that will enable the detection of a disease in its early stages as well as to find applications for monitoring its course, making the appropriate choice of therapy, controlling the body's response to the implemented treatment and the differentiation of diseases with a similar clinical view or non-specific symptoms.

The discovery of clusterin (CLU) presence in almost all body tissues and fluids and the multidirectional biological role which it plays in the human body have become the basis of many studies concerning the use of this glycoprotein as a potential new biomarker in the diagnosis of numerous human diseases. Clusterin, also known as apolipoprotein J (ApoJ), is a glycoprotein that exists in two forms: the highly glycosylated secretory form of clusterin (sCLU) and its intracellular nuclear form (nCLU), which is still not well characterized [1, 2]. Secretory clusterin is considered a molecule with the properties of a chaperone protein, and its activity depends on the degree of glycosylation [3, 4]. Clusterin binds to specific cell surface receptors and thus mediates many biological processes such as endocytosis and apoptosis [5, 6]. Although the biological role of clusterin remains to be fully understood, it is undeniable that increased clusterin concentrations are associated with homeostasis disorders in many pathophysiological conditions, including atherosclerosis [7], obesity [8], diabetes [9], and Alzheimer's disease (AD) [10, 11]. The analysis of scientific reports on changes in the concentration of clusterin and the degree and/or profile of its glycosylation will allow us to check whether CLU can be an additional diagnostic marker helpful in the diagnosis of various diseases.

Our review is based on literature research performed in the PubMed and Google Scholar databases using search terms and their combinations, including clusterin, clusterin glycosylation, glycoprotein glycosylation, cardiovascular diseases, metabolic diseases, male infertility, cancer, and neoplasm. As a result, over 7000 entries, published from 1983 to the present, mostly in English, were found. Finally, the 165 items, mainly from original papers, which in our opinion seemed to be most useful for our investigation, were selected. Investigations on cell lines and animal models are also covered in the present review, as their goal is usually to understand mechanisms of reactions and their interrelationships that reflect those occurring in the human body. This article comprises a review of the recently available literature concerning variability in clusterin molecular structure, with a focus on particular changes in its profile and degree of glycosylation occurring in human tissues and body fluids, analyzed in the context of the utility of these characteristics as the potential diagnostic biomarkers of selected civilizational diseases.

## 2. Clusterin

The name of the discussed glycoprotein was coined in 1983 by Blaschuk and coworkers, who identified a high-molecular-weight protein in the fluid of a ram testicle and named CLU for its ability to cluster Sertoli cells [12]. The human clusterin (apolipoprotein J, ApoJ) is also sometimes called a complement-lysis inhibitor (CLI) or a complement-associated protein SP-40,40, but these names are not commonly used. Clusterin is involved in many biological processes such as cell adhesion, cell membrane restoration, complement system inhibition, sperm maturation, lipid transport, and apoptosis [13]. For example, human seminal plasma proteomic analysis has demonstrated the existence of at least 43 isoforms of sCLU, the presence of which is probably related to the maintenance of homeostasis in the body [14].

### 2.1. Clusterin Structure

**Figure 1:**
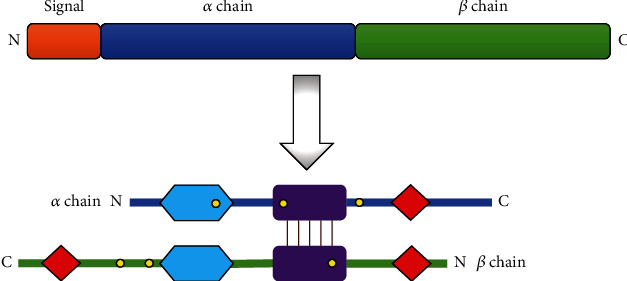
A scheme of the secretory isoform of clusterin. The purple color shows cysteine-rich centers connected to each other by five disulfide bounds. Two coiled-coil *α*-helices are indicated in blue, while three amphipathic *α*-helices are shown in red. N-glycosylation sites are indicated by yellow dots. Self-modification based on Wilson and Easterbrook-Smith [18] and Fini et al.[19].

Clusterin is a heterodimeric glycoprotein with a molecular weight of about 75-80 kDa, encoded by a single gene located on the short arm of chromosome 8, near the lipoprotein lipase gene locus. The primary clusterin polypeptide chain, composed of 449 amino acids, undergoes proteolytic cleavage, resulting in the formation of alpha and beta chains. The chains are linked by five disulfide bonds and form a two-chain, antiparallel glycoprotein structure. The core of the clusterin molecule is surrounded by three amphipathic *α*-helices and two *α*-helices with a coiled-coil structure. About 30% of the molecular weight of clusterin is constituted by N-linked glycans attached to the protein structure at six specific sites. Three of them are located within the *α*-chain (*α*64Asn, *α*81Asn, and *α*123Asn) and the rest on the *β*-chain (*β*64Asn, *β*127Asn, and *β*147Asn) [15, 16]. Clusterin is present in all human body fluids: urine, blood plasma, cerebrospinal fluid, semen, and even breast milk. It possesses the ability to form oligomers and may also interact with many ligands, forming complexes of various diameter and mass. The structure of this glycoprotein is flexible due to the presence of both hydrophilic and hydrophobic regions [17]. The schematic structure of secretory clusterin is presented in Figure 1.

### 2.2. Clusterin Properties

**Figure 2:**
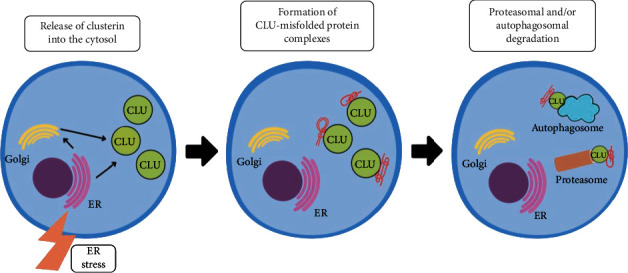
Schematic representation of intracellular role of CLU in misfolded protein degradation. CLU: clusterin; ER: endoplasmic reticulum. Self-modification based on Satapathy and Wilson [29] and Nizard et al. [30].

**Figure 3:**
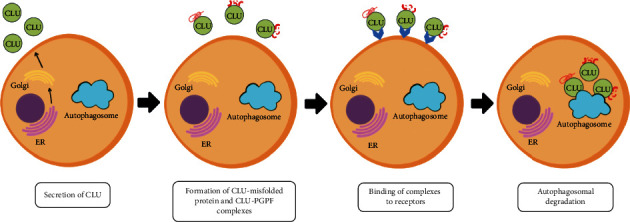
Schematic representation of extracellular role of CLU in misfolded protein degradation. CLU: clusterin; ER: endoplasmic reticulum; PGPF: plasmin-generated protein fragments. Self-modification based on Wyatt et al. [31].

Clusterin shows extracellular chaperone properties, binding to proteins damaged by various factors, such as high temperature, oxidative stress (OS), or chemical reducing compounds. The combination of clusterin with misfolded proteins is ATP-independent and leads to the formation of high molecular weight soluble complexes, which are then removed by endocytosis and lysosomal degradation. This mechanism prevents the formation of pathological aggregates and may take place both inside or outside the cells [14, 20]. Clusterin, released into the cytosol after posttranslational ER modification, interacts with misfolded proteins, forming complexes, which are subsequently degraded in proteasomes and/or autophagosomes (Figure 2). CLU secreted into extracellular matrix (ECM) generates complexes not only with misfolded proteins, but also with plasmin-generated protein fragments (PGPF), which are formed due to the action of the circulating protease, plasmin. Such complexes bind to specific cell receptors, are internalized by receptor-mediated endocytosis, and then transported to autophagosomes for degradation, as shown in Figure 3 [21]. The effect of clusterin as a chaperone protein is enhanced under the environmental conditions below pH=7 that occur during local acidosis resulting from tissue damage or inflammation. This is due to the increased exposure of clusterin's hydrophobic regions under conditions of lowered pH [22]. Stewart et al. have hypothesized that the presence of numerous hydrophilic carbohydrate groups in the clusterin structure enables its chaperone activity [23]. To confirm this hypothesis, the authors compared the structure and function of the native and deglycosylated forms of human CLU. It was proven that although the deglycosylation of clusterin does not cause significant changes in the secondary protein structure, it raises the molecules' tendency to aggregation [23]. Disturbances in the function of clusterin as a chaperone protein contribute to the development of storage diseases such as amyloidosis, atherosclerosis, Alzheimer's or Creutzfeldt-Jakob disease [14, 20]. Blood plasma CLU combines with apolipoproteins A and E to form the HDL (high density lipoprotein) molecule so that it can participate in the transport of cholesterol from peripheral tissues to the liver. Clusterin promotes the export of cholesterol and phospholipids from foam cells, which are characteristic of atherosclerotic lesions [24]. Clusterin is an integral component of the plasma-present C5b-9 complex which takes part in the initiation of the complement cascade. Studies on the role of clusterin in the human body have shown its inhibitory effect on membrane attack complex (MAC) production, which prevents the lysis of cell membranes, thus protecting cells from the uncontrolled action of MAC, which leads to cell apoptosis. The presence of CLU may prevent uncontrolled attack on the cell membrane by proteins that are a part of the complement system, but also may protect the cell against other damaging factors originating from the extracellular environment [17]. It is worth paying attention to the bipolar action of clusterin. It has been documented that a decrease in CLU concentration in the heart muscle tissue is associated with reduction in the degree of its damage and the inhibition of the neuronal apoptosis process under conditions of hypoxia or ischemia. On the other hand, the decreased CLU concentration observed in immune myocarditis leads to an increase in myocardial tissue damage [16].

In the male reproductive tract, clusterin is produced by Sertoli cells and then attaches to the cell membranes of spermatids and mature sperm, taking part in the process of spermatogenesis. There are two main forms of clusterin: anti-apoptotic secretory (sCLU) and nuclear (nCLU) with proapoptotic properties. Secretory clusterin protects cells against toxic factors that activate apoptosis due to the suppression of p53 and Bax protein (Bcl-2-associated X protein). Inhibition of the secretory clusterin gene expression leads to an increase in the apoptotic index [25]. The induction of clusterin gene expression was first associated with cell apoptosis in rat prostate regression. The effect of the degree of clusterin expression on cell survival and death was investigated [26]. It was reported that in human prostate cells, overexpression of clusterin provides protection against the action of TNF-*α*, which induces apoptosis [27]. The results of the above studies suggest that nuclear clusterin may play a cytoprotective role against epithelial cells. At this stage of the investigation, there is no clear evidence that clusterin is directly involved in the mechanism of programmed cell death or that the induction of CLU gene expression is a secondary process to apoptosis. To assess the significance of CLU in the apoptosis process, monoclonal antibodies that recognize the wild-type clusterin molecule and a unique isoform related to apoptosis were used [28]. Results of a study concerning the impact of apoptosis factors on clusterin present in Michigan Cancer Foundation 7 cells (MCF-7) confirmed that significant changes in the biogenesis of clusterin occur in the process of apoptosis and result in the appearance of a non-glycosylated CLU isoform in the cell nucleus, which initiates DNA fragmentation, proving that nCLU has proapoptotic properties [26].

The concentration of clusterin in human seminal plasma is considered a prognostic factor for regeneration of sperm production in azoospermic patients (nonobstructive azoospermia due to inhibition of sperm production). Therefore, the analysis of clusterin concentration changes in seminal plasma may be an important element in the diagnosis of male infertility linked with lack or lowered count of sperm [25]. It has been shown that antisense mutations in the CLU gene, resulting in the silencing of gene expression, may increase the chemosensitivity of prostate cancer cells, which is probably caused by blocking the antiapoptotic properties of clusterin [16].

### 2.3. Clusterin as DC-SIGN Ligand

**Figure 4:**
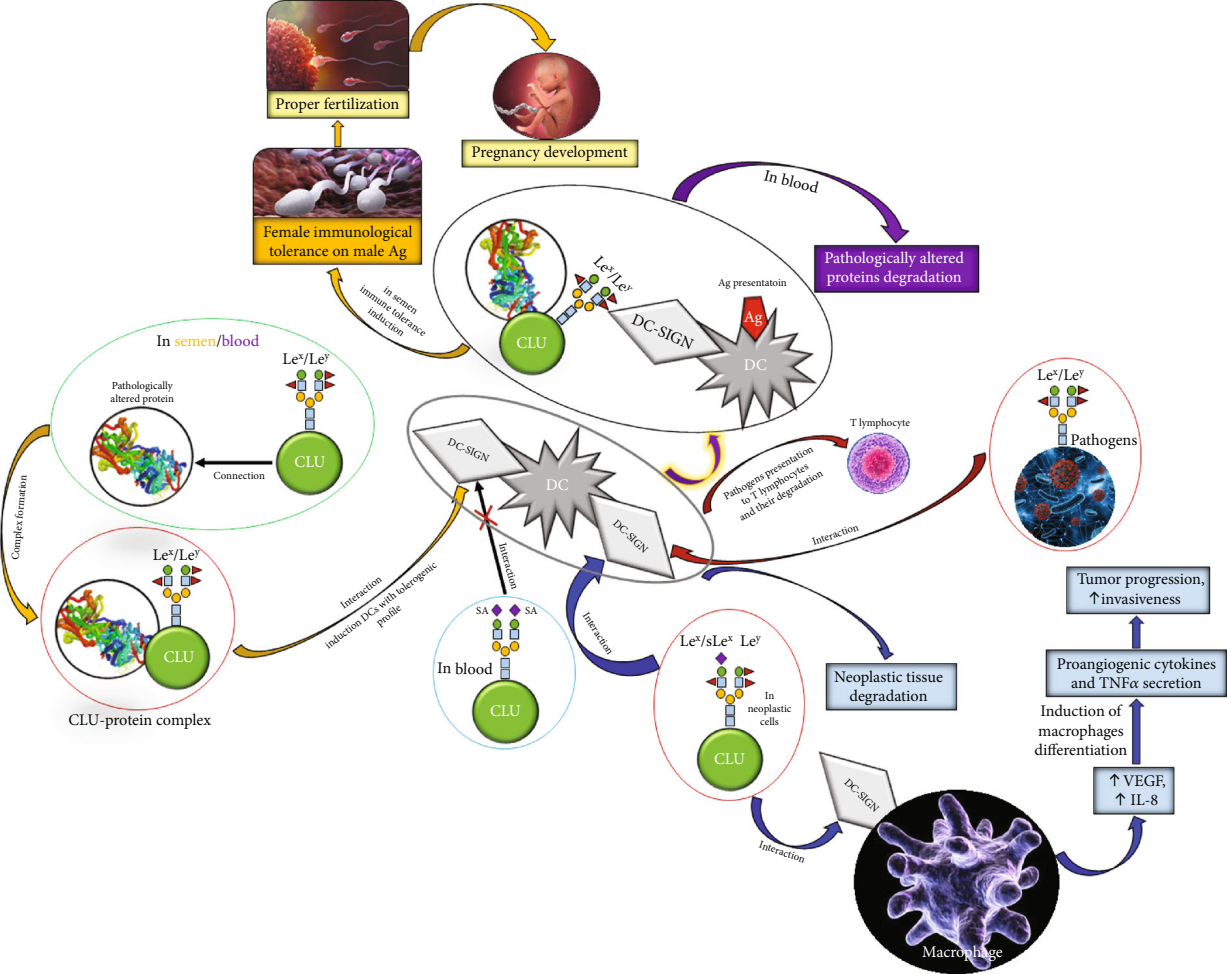
A scheme of the main processes occurring as a result of the interaction between CLU and DC-SIGN. Ag: antigen; CLU: clusterin; DC: dendritic cell; DC-SIGN: dendritic cell–specific intercellular adhesion molecule-3-grabbing non-integrin; IL-8: interleukin 8; VEGF: vascular endothelial growth factor; Le^X^/Le^Y^: Lewis^X^/Lewis^Y^ oligosaccharide structures; SA: sialic acid. Yellow arrows: CLU participation in reproduction process; purple arrows: CLU activity in the degradation of pathologically altered proteins in blood; blue arrows: CLU participation in neoplastic tissue degradation and/or tumor progression; red arrows: CLU activity in pathogens' degradation.

The clusterin present in seminal plasma contains highly fucosylated Lewis^x^ (Le^x^) and Lewis^y^ (Le^y^) structures, which are responsible for its ability to bind to DC-SIGN (dendritic cell–specific intercellular adhesion molecule-3-grabbing non-integrin), a type C lectin receptor selectively expressed on dendritic cells (DCs) [32, 33]. In contrast, glycans of serum CLU contain mainly sialylated structures without Lewis-type sugar ligands [15, 33], and consequently blood plasma clusterin does not bind to the DC-SIGN receptor [32]. Seminal plasma CLU not only possesses chaperone activity similar to the blood plasma clusterin, but also addresses stress-altered proteins to the DCs via DC-SIGN. The complexes composed from clusterin and pathologically altered proteins are then processed inside the DCs and presented on their surface in antigen form (Figure 4). It is hypothesized that this mechanism may play an important role in the maintenance of immunological tolerance to paternal antigens in the fertilization process and proper pregnancy development. It should be underlined that the acquisition of female tolerance to the partner's antigens requires an active immune response that includes the participation of both DCs and regulatory T lymphocytes [34, 35]. Induction of tolerance to the semen antigens requires interaction with DCs. Steinman et al. [36] suggested that clusterin present in the seminal plasma may play a role in this interaction, promoting the endocytosis of antigens in semen by DCs via DC-SIGN. One of the most important functions of the seminal plasma clusterin is not to remove misfolded proteins from the extracellular space, but to direct these proteins to the DCs for antigen presentation, which results in immune tolerance induction [36]. The interaction of clusterin with DCs via DC-SIGN induces differentiation of dendritic cells with a tolerogenic profile (Figure 4). This process enables maternal tolerance towards male alloantigens [37].

#### 2.3.1. The Importance of Clusterin Expression in Pathophysiological Conditions

The physiological concentration of blood plasma clusterin is about 100 *μ*g/mL, and in semen, it is at least 20 times higher, assuming values in the range of 2-10 mg/mL [38]. However, the particular values obtained for human seminal plasma vary between different authors, which may be the result of using different methods for determination of CLU concentration, differing in sensitivity. In contrast, mean seminal plasma CLU levels obtained by Fukuda et al. [39] expressed in ng/mL (from 14.48±9.74 ng/mL in nonobstructive azoospermic patients, up to 48.31±38.59 ng/mL in the control group) were comparable with findings of Janiszewska et al., who reported the following mean seminal plasma CLU levels in infertile normozoospermic, teratozoospermic, asthenoteratozoospermic, and oligoasthenoteratozoospermic groups: 36.46 ng/mL, 33.08 ng/mL, 29.43 ng/mL, and 66.59 ng/mL [33]. On the other hand, the CLU levels in the blood sera of infertile patients were as follows: 21.53 *μ*g/mL, 37.53 *μ*g/mL, 38.25 *μ*g/mL, and 36.73 *μ*g/mL for normozoospermic, teratozoospermic, asthenoteratozoospermic, and oligoasthenoteratozoospermic men, respectively [33]. An increase in concentration of clusterin can be observed *inter alia* in the course of type 2 diabetes, ischemic heart disease, prostate cancer, and hepatocellular carcinoma (HCC) [40–42]. Determination of the particular role of CLU in various diseases based on changes in CLU gene expression is difficult. It is assumed that the expression of the CLU gene promotes cell survival, which may have two effects in the context of the whole organism, either beneficial, promoting neuronal survival by combating toxic agents, or detrimental, enabling tumor cells to survive. It was noticed that increased expression of the CLU gene occurs in treatment-resistant neoplasms [43]. CLU binds to low density lipoproteins such as very low density lipoproteins (VLDL) and low density lipoprotein-related protein 2 (LRP2) [44, 45]. The combination of clusterin with LRP2 induces the activation of serine-threonine kinase, also known as protein kinase B (PKB), promoting cell survival [45], and inhibits the transduction of proapoptotic signals through interaction with cell surface receptors [46].

To conclude, the issue of CLU gene expression under pathophysiological conditions, as well as its concentration in *inter alia* human blood serum and seminal plasma, is not completely defined. No simple relationship between CLU level and the expression of the CLU gene was found, and thus further studies concerning this subject are needed.

#### 2.3.2. The Importance of Clusterin in the Formation of Neoplastic Metastases

Clusterin can play a variety of functions in carcinogenesis and tumor invasion processes [47]. First and foremost, it enables neoplastic cells to survive in places distant from the primary tumor, which makes it possible for them to form metastases [48]. Increased expression of CLU has been demonstrated in the metastatic or cancerous cells of colon, bladder, and hepatocellular carcinoma [49–51]. Miyake et al. introduced CLU complementary DNA into human renal cell carcinoma cells, which do not express a detectable level of clusterin expression, and their findings suggest that clusterin overexpression prolongs cell survival under unfavorable conditions in the metastatic process, resulting in the enhanced metastatic potential of renal cell carcinoma, which confirms the positive effect of clusterin on neoplastic cell migration [52]. The results of the above studies, as well as the studies carried out by Chou et al. [53], proved that the increase in the expression of clusterin is associated with the degree of tumor invasiveness. Knowledge of the mechanism of metastasis formation induced by increased expression of clusterin has been used to create new anticancer therapies aimed at metastasis formation inhibition and tumor growth blockage [54]. Shiota et al. observed that overexpression of CLU is associated with low tumor histological differentiation and high advancement in clinical TNM classification (tumor node metastasis scale) [55]. The ability of clusterin to promote tumor invasion is based on epithelial-mesenchymal transition (EMT) induction [56], a process by which epithelial cells transform into mesenchymal tissue, losing the ability to adhere and gaining the possibility of moving to other parts of the body [56].

It has also been documented that clusterin increases the resistance of ovarian cancer to treatment, preventing drug interactions with neoplastic cells, thus preventing the induction of apoptosis and, consequently, the fight against cancer [57]. It has been proven that sCLU enhances the neoplastic process by facilitating Ku70 binding to the apoptotic protein Bax, as a result of which the Bax protein cannot reach the outer membrane of the mitochondria and transmit the signal to direct the cell to the apoptotic pathway [58].

Over 90% of fatal cancer cases are associated with the appearance of metastases, which are linked with lack of effective therapy—surgical treatment with adjuvant therapy is successful only in the case of primary, well-defined tumors [59, 60]. Still, many fundamental questions regarding the development of neoplastic metastases remain unanswered. It has been suggested that the determination of CLU concentration in neoplastic tissue or serum may be a potential diagnostic marker of early neoplastic metastases [61]. The results of the latest preclinical studies showed that inhibition of clusterin expression delays the development of metastases and increases sensitivity to cytotoxic chemotherapy, significantly improving the survival rate of cancer patients [62].

In summary, CLU enables neoplastic cell survival, as proven in particular in cases of colon, bladder, ovarian, and hepatocellular neoplasms. High CLU expression is also related to tumor invasiveness as well as metastasis formation, especially within the EMT mechanism. There is also some information about the potential role of CLU in treatment resistance in ovarian cancer. On the other hand, it has also been suggested that in some neoplasms, CLU concentration in serum and/or tissue may become an early metastasis biomarker.

## 3. The Biological Role of CLU Glycosylation

### 3.1. The Dependence of CLU Function as a Chaperone on Glycosylation

Clusterin is one of the few known extracellular chaperone proteins. Similarly to other chaperones, the mRNA of CLU undergoes positive regulation during heat shock [63, 64]. The organism's response to the conditions created during heat shock is reflected by increased production of proteolytic enzymes, detoxification proteins, and chaperones [65]. This is a form of defense against negative effects of UV and ionizing radiation or oxidative factors which lead to the formation of reactive oxygen species (ROS), resulting in cumulation of abnormally folded proteins [66]. Such interference disrupts protein homeostasis and may cause toxic stress. CLU, playing the chaperone role, prevents oxidative stress effects through binding with the proteins undergoing denaturation, and thus preventing their accumulation, as well as allowing them to be removed from the extracellular space [22].

Apart from extracellular secretory CLU, the cytoplasm of cells undergoing OS contains a small amount of another isoform of CLU, mainly non-glycosylated, which did not undergo proteolytic degradation, forming a dimeric structure consisting of alpha and beta subunits as in the case of sCLU formation [64]. Rohne et al. in their study [4] investigated whether intracellular CLU (iCLU) possesses chaperone properties. The authors reported that non-glycosylated iCLU cannot perform its chaperone activity. In contrast, the proper composition of antennary oligosaccharides in sCLU glycans was not obligatory to maintain the chaperone activity of this glycoprotein, whereas proper core oligosaccharide structure was crucial to maintaining its chaperone activity [4]. Debure et al. demonstrated the high sensitivity of iCLU to reducing factors, proving that intracellular forms of CLU only become active under oxidative stress conditions or in subcellular regions with altered reduction potential, e.g., in mitochondria [67]. It may be assumed that, in terms of homeostasis disturbance due to an inflammation process or other pathological conditions, iCLU may exhibit chaperone activity [67]. The results of the aforementioned research prove that CLU glycosylation is crucial to the maintenance of its function and proteolytic cleavage is the key to chaperone activity exhibition by CLU under reducing conditions, e.g., in atherosclerosis [4].

It has been proven that sCLU, as one of the few well-known extracellular chaperones, with a proper composition of core oligosaccharide structure can perform chaperone activity. Another intracellular, non-glycosylated isoform of CLU is formed in response to oxidative stress. Resistant to proteolytic cleavage, its chaperone activity is possible only under oxidative stress conditions or in subcellular regions with altered reduction potential.

### 3.2. The Impact of CLU Deglycosylation on the Cytotoxicity Development

Apart from sCLU, there are several variants of intracellular clusterin, which differ in their molecular weight. In cells stimulated by various factors, such as those that induce apoptosis, TGF-*β* (transforming growth factor *β*), TNF-*α* (tumor necrosis factor *α*), and ionizing radiation, the formation of reduced, nuclear forms of CLU with a molecular weight of 43-55 kDa were observed [26, 68]. Elevated nCLU expression constitutes a proapoptotic factor. Moreover, a fully non-glycosylated iCLU variant with molecular weight of 60 kDa was found in the mitochondria of human cancer cells, promoting neoplasm progression through disruption of the pro-apoptotic activity of the Bax protein [46, 69]. The differences in molecular weights observed in iCLU forms were explained by changes in the process of posttranslational modifications caused by various factors [26].

CLU is sensitive to homeostasis disruption in the endoplasmic reticulum (ER) area, where the glycosylation process takes place [70]. Pathological factors present in the ER area disrupt the glycosylation process and lead to structural instability and/or impairment of intra- and extracellular clusterin function [71]. Kang et al. conducted studies to assess the effect of glycosylation on the accumulation of iCLU [72]. The results of these studies provide important information on the molecular basis of pathological conditions caused by impaired CLU biogenesis. To determine whether clusterin responds to stress factors within ER, researchers assessed CLU expression in cell cultures exposed to three different stress inducers of ER: dithiothreitol (DTT), thapsigargin (Tg), and tunicamycin (Tm). Western blot analysis, performed after previous electrophoresis under reducing conditions, showed the existence of two different forms of CLU: one with a mass of 60 kDa (probably a native CLU molecule) and the other with a mass of 40 kDa, which appeared to be an *α* subunit of the native CLU form. Moreover, DTT and Tg lowered the expression of both CLU forms, and Tm initiated the synthesis of the non-glycosylated forms with reduced solubility that were accumulated in ER [72]. The results of a study performed by Kang et al. [72] also suggested that disturbed CLU glycosylation leads to the accumulation of its abnormal molecules in ER, causing cytotoxicity with simultaneous activation of unfolded protein response (UPR)—a process of degradation of misfolded proteins which takes place in the proteasome. It has been documented that the oligosaccharides of CLU N-glycans are important determinants preventing CLU misfolding and aggregation in ER as well as N-glycans deficiency in CLU, which results in its accumulation in ER and induces cytotoxicity, which may be the cause of various diseases. The authors suggested that the discussed disorders are involved in the pathomechanism of cellular dysfunction in slowly progressive neurodegenerative diseases caused by excessive pathological protein accumulation [72].

In summary, it was documented that elevated levels of glycosylated nuclear CLU isoform have a proapoptotic effect. On the other hand, deglycosylation of CLU promotes its antiapoptotic properties. Moreover, the fully non-glycosylated CLU variant disturbs the proapoptotic activity of Bax, leading to neoplasm progression. Abnormal, disturbed, CLU glycosylation process leads to the accumulation of its abnormal form in the ER. It has been suggested that the aforementioned mechanisms lead to neurodegenerative diseases associated with excessive abnormal protein accumulation.

### 3.3. The Importance of Clusterin Glycosylation for Regulating the Immune System in Human Reproduction

Human seminal plasma proteome analysis expanded our current knowledge concerning the mechanisms of the fertilization process. Posttranslational modifications, including glycosylation, have a strong effect on cell-cell interactions and interactions between the inside of the cell and surface proteins, or proteins present in secretory fluids. It has been documented that the sialylated form of Le^x^ structures is the most important carbohydrate ligand present on the oocyte cell membrane, mediating sperm binding [73]. Another example documenting the importance of the glycosylation process is glycodelin, which in its glycosylated form is responsible for the acrosomal reaction [74]. Glycoproteins also play an important role in maternal immune response modulation, and glycans present on the sperm surface suppress the activity of maternal NK (natural killers) cells [75]. Le^x^ and Le^y^ oligosaccharide structures present in seminal plasma glycoproteins also exhibit an inhibitory effect on the female immune system [76]. Analysis of the seminal plasma glycome has made it possible to divide N-glycans into three main classes: high mannose; fucosylated bi-, tri-, and tetra-antennary Le^x^ and/or Le^y^ type oligosaccharide structures, and fucosylated and sialylated bi-, tri-, and tetra-antennary N-glycans [77].

A study by Saraswat et al. [78] enabled to establish the glycosylation profile and the glycan structure of glycoproteins present in seminal plasma, creating an opportunity to determine the role of glycoproteins in immune system modulation and gamete interaction. In subsequent studies, the same authors proved that all clusterin N-glycans were of the complex type, of which: 12 were sialylated and others, at the end of sugar antennas, contained an exposed galactose residue; 21 glycans contained Le^x^/Le^a^ type oligosaccharide structures; another 3 had a sugar structure characteristic for the H antigen of the blood group; and one contained Le^y^/Le^b^ oligosaccharide structures [78]. The role of seminal plasma proteins in the fertilization process has not been fully understood, and their biological function apparently includes participation in sperm protection from premature capacitation and sperm transport inside the cervix [79], as previously shown for glycosylated glycodelin, which, contrary to its non-glycosylated counterpart, inhibits the premature capacitation process [80].

A recent study by Janiszewska et al. [33] concerning blood serum and seminal plasma CLU concentration and fucosylation analysis indicates that serum CLU concentrations, as well as the expression of core fucose and antennary fucose *α*1,2-linked in CLU glycans, differ between an infertile normozoospermic group and groups of patients with sperm disorders and seem to be good markers for differentiation of normozoospermic men from those with sperm abnormalities. The authors [33] also suggest that disturbances in sperm count, motility, and morphology are not the only causes of male infertility. Moreover, the lack of similarities in levels of blood serum and seminal plasma CLU, as well as in fucose expression in CLU glycans, is probably due to the different mechanisms leading to CLU glycosylation in both body fluids analyzed by the authors [33].

Human clusterin is known as a ligand for DC-SIGN [78]. DC-SIGN has the ability to bind the Le^x^/Le^y^ type oligosaccharide structures present on the surface of many pathogens and enables dendritic cells to recognize surface antigens of pathogens and present them to T lymphocytes (Figure 4) [81]. Glycosylation analysis of seminal CLU revealed that it contains Le^x^/Le^a^ and/or Le^y^/Le^b^ types of oligosaccharide structures at the Asn374, Asn354, and Asn86 sites, which may indicate that clusterin is involved in immunomodulation in seminal plasma [78]. It has been proven that the major carbohydrate structure in N-glycans of zona pellucida (ZP), participating in sperm-oocyte interaction, is sialo-Le^x^ type, a well-known selectin ligand. Thus, any alterations in the zona pellucida glycoepitopes, as well as sperm surface glycans, may cause an unsuccessful fertilization process [73].

CLU is a very important glycoprotein in human semen that takes part in sperm maturation and thus in the fertilization process. Seminal plasma CLU, but not blood serum clusterin, expresses an extreme abundance of fucosylated glycans. These glyco-motifs enable seminal plasma clusterin to bind DC-SIGN with very high affinity. By inducing the endocytosis of stress-damaged proteins by DCs via DC-SIGN, clusterin present in seminal plasma may promote female tolerance to seminal antigens (Figure 4).

Sialo-Le^x^ oligosaccharide structures on the oocyte membrane are considered the main carbohydrate ligands mediating sperm binding. Moreover, it has been proven that not only Le-type oligosaccharide structures present in the glycan part of seminal plasma CLU are of crucial importance in the context of reproduction, but also the expression of core fucose differs between patients with abnormal semen parameters and those without sperm disorders. Further analyses concerning changes in glycosylation profile and degree of seminal plasma and serum CLU may shed new light on the complex mechanisms of proper fertilization processes.

### 3.4. The Importance of Fucosylated Clusterin in Breast Cancer

The disturbance of blood plasma clusterin expression has been documented in many types of cancer, including breast cancer [82]. Increased expression of blood plasma CLU usually correlates with negative prognosis, disease recurrence, or the development of resistance to treatment [83]. Chen et al. [84] have reported significantly higher serum CLU levels in invasive breast cancer patients in comparison to the healthy controls. Moreover, strong correlations between CLU concentration and clinical tumor stage, lymph node metastasis, shorter overall survival, and disease-free survival were observed. Patients after surgery treatment revealed lower CLU concentrations when compared to the presurgery stage. The diagnostic sensitivity and specificity of serum CLU level determinations in this study were 82.26% and 73.46%, respectively [84]. While the expression of CLU in the cytosol of neoplastic cells inhibits their apoptosis, promoting tumor growth and the development of resistance to chemotherapy, nCLU shows proapoptotic properties [1]. Secretory CLU is present in the extracellular space and body fluids, but its influence on the microenvironment of the developing tumor is poorly understood. It is worth underlining that CLU glycans in neoplastic tissues possess different glycan motives than those occurring in healthy tissues, which is often termed “abnormal glycosylation” [85].

Changes in the expression of Le structures are some of the most common alterations in protein glycosylation in neoplastic processes [86]. Neoexpression of sialo-Le^x^ structures makes the tumor cells capable of binding to the endothelial selectins, resulting in the formation of metastases [87]. Merlotti et al. analyzed the expression of fucosylated clusterin in breast cancer cells [88]. A tumor fragment and a piece of tumor-free tissue adjacent to the tumor were collected from each of 21 patients. Subsequent immunohistochemical analysis revealed the CLU expression, which enabled researchers to observe its presence both in the cytoplasm of tumor cells and in the apical parts of the cells of healthy tissues. The ELISA test (enzyme-linked immunosorbent assay) enabled CLU level quantification in tumor and healthy tissue samples, but there were no significant differences in clusterin concentrations between the compared groups [88]. The changes in the glycosylation profile of neoplastic tissues have been well documented, and the increased fucosylation degree of glycoproteins of neoplastic cells is probably associated with their increased invasiveness and ability to metastasis [86]. Merlotti et al. documented that breast tumor tissues are characterized by a higher expression of fucosylated CLU containing Le^x^ and Le^y^ structures in comparison to tissues without tumor transformation [88]. The authors reported that the fucosylated CLU of neoplastic cells, in contrast to the CLU present in healthy cells, has the ability to bind to DC-SIGN, proving that CLU of breast cancer cells contains fucosylated glycans of Le^x^ and/or Le^y^ type, enabling effective binding to DC-SIGN. Considering the fact that fucosylated clusterin effectively interacts with macrophages by interacting with DC-SIGN, the authors analyzed the effect of this interaction on macrophage phenotype and function. They conducted a study in which monocytes were cultured with M-CSF (macrophage colony-stimulating factor) for 5 days in the absence or presence of fucosylated CLU isolated from seminal plasma. It has been observed that the addition of fucosylated clusterin to the cell culture significantly increased the expression of angiogenic factors such as vascular endothelial growth factor (VEGF) and interleukin 8 (IL-8), suggesting that fucosylated CLU induces the differentiation of macrophages into cells that secrete proangiogenic cytokines and TNF-*α*. It has also been suggested that the fucosylated form of CLU produced by luminal breast cancer cells might play an important role in tumor advancement (Figure 4) [88].

It has been documented that CLU levels are associated with breast cancer development and correlate with tumor stage, lymph node metastasis, and disease-free survival as well as overall survival. After surgery, lowered CLU concentrations in comparison to the presurgery state were observed. Several studies have reported that neoplastic tissues express glycan motives distinct from those in healthy tissues. Increased CLU glycans fucosylation and sialo-Le^x^ expression is associated with elevated invasiveness and metastasis formation, via interactions with DC-SIGN, leading to pathological angiogenesis, as shown in Figure 4.

### 3.5. The Importance of Clusterin Glycoforms in Colorectal Cancer

Understanding the function of clusterin in carcinogenesis has been the subject of research for several decades. Chen et al. observed the presence of increased CLU concentrations in neoplastic cells among patients with human colorectal cancer (CRC) at an early stage of its development, i.e., with early intestinal lesions, benign polyps, or adenocarcinoma [89]. Pucci et al. [90] proved that increased sCLU expression in the cytoplasm of neoplastic cells also applies to patients with malignant colon tumors in the course of metastases to the lymph nodes. Many factors indicate that clusterin is involved in the mechanism of regulation of contrary processes such as cell survival and apoptosis [91], which has been observed both in colon cancer [90, 92] and other types of cancer, e.g., bladder cancer [93], kidney cancer [52], and prostate cancer [94]. It has been shown that increased expression of sCLU in neoplastic cells leads to the development of resistance to the cytotoxic pharmaceuticals used in anticancer therapy, which aim to induce apoptosis of cancer cells [95].

Chen et al. observed the presence of a correlation between the expression of an intracellular CLU isoform and tumor progression, which led to clusterin being considered a potential prognostic and predictive colon cancer marker [89]. In their study on tissue specimens, Artemaki et al. [50] showed that patients with significantly higher levels of CLU mRNA in tumors were at higher risk of recurrence or death and that the expression of CLU mRNA increased together with tumor growth and disease progression. Based on the results obtained, the authors suggested that the high levels of CLU mRNA may be usable as an adverse prognostic biomarker for disease-free survival and overall survival in colorectal cancer [50]. Rodríguez-Piñeiro et al. undertook studies related to the expression of blood plasma CLU isoforms in patients with CRC to determine whether the CLU molecule present in the blood plasma of CRC patients is altered, compared to the CLU present in the blood plasma of healthy subjects [92]. Increased concentration of sCLU and decreased expression of the deglycosylated form of nCLU were associated with increased viability of cancer cells and the possibility of metastasis [92]. Chromatographic analysis using ConA (*Concanavalin A* agglutinin, binds multimannose N-glycans, complex and hybrid type), combined with two-dimensional gel electrophoresis (2D-PAGE), enabled the isolation of two serum fractions: FI, rich in O-glycosylated proteins, but without N-glycans, and FII fraction, rich in N-glycoproteins. Anti-CLU antibodies were added to the obtained fractions and to the nonfractionated serum samples, which demonstrated the presence of heterodimeric, glycosylated 70-85 kDa clusterin molecule in the native serum samples and in the FII fraction [92]. In the FI fraction, obtained from the sera of patients with CRC, the presence of CLU with a molecular weight of about 40 kDa was additionally demonstrated. This was most likely a CLU molecule with reduced N-glycan expression (the molecular weight of the double-stranded CLU protein chain is no more than 30 kDa). As a result of deglycosylation of this 40-kDa molecule, a protein with a molecular mass of about 28 kDa was obtained, confirming previous assumptions. Structural analysis of specific CLU isoforms, isolated from the serum of patients with CRC, showed the presence of significant differences in comparison to CLU isoforms present in the control serum. In CRC patients, increased expression of five isoforms in the 40-kDa band in the FI fraction and one isoform in the FII fraction was observed, while the isoforms present in the 40-kDa band probably corresponded to the isoform detected by Pucci et al., who observed increased expression of cytoplasmic glycosylated CLU in the advanced stage of cancer with metastases to the lymph nodes [90]. This isoform has also been shown to be released into the extracellular space [90]. Rodríguez-Piñeiro et al. [92] also noted that the CLU isoforms in the 40-kDa band, present in the FI fraction isolated from the sera of CRC patients, were highly glycosylated. However, this glycosylation was probably abnormal, while their molecular mass corresponded to the molecular mass of CLU (approximately 40 kDa) described by Pucci et al. [90]. The results of studies performed by Rodríguez-Piñeiro et al. clearly show that the analysis of expression of individual CLU isoforms in the blood plasma of patients with CRC may become an effective diagnostic tool for this type of cancer [92]. Taking into account that there is still a great need for new, specific, and sensitive markers that would find application for CRC diagnosis and monitoring of its treatment, it seems justified to undertake further research on the use of CLU isoforms expression analysis for this purpose.

Several studies confirmed elevated CLU levels in neoplastic cells of colorectal cancer. The correlation between iCLU and tumor progression has also been demonstrated; thus, clusterin may become a prognostic and predictive CRC biomarker. As in the case of other tumors, the role of CLU is associated with inhibitory activity on the Bax protein. Some analysis concerning CLU structure and glycosylation revealed that patients with colorectal cancer are characterized by an increased CLU glycosylation, which was probably abnormal. Further glycomic studies will help to improve our knowledge about the molecular mechanisms concerning this issue.

### 3.6. The Importance of Secretory Clusterin Glycosylation in Hepatocellular Carcinoma

Hepatocellular carcinoma is one of the most common causes of death among cancer patients worldwide [96]. Factors involved in the development of HCC include chronic hepatitis B (HBV) or C (HCV), nonalcoholic steatohepatitis, and aflatoxin B1 poisoning. The prognosis of HCC patients depends on early diagnosis and the effectiveness of the selected treatment method. Despite the progress of therapy, the prognosis of patients is still poor, due to the frequent relapse of the disease after surgery and the risk of metastases. Therefore, selecting markers whose expression level would be closely related to the clinical advancement of HCC and enable the monitoring of metastasis formation is necessary to increase the chances of patients' survival [97].

Monitoring the course of liver disease, including the control of tumor cell growth, is currently based on the determination of *α*-fetoprotein (AFP) concentration or analysis of the degree of its glycan core fucosylation. However, AFP synthesis is associated not only with liver cancer but also with other non-oncological pathologies of this organ [98], and its secretion is not observed in all cases of HCC [99]. Therefore, more specific biomarkers useful in the diagnosis of HCC are still being sought.

In the liver, sCLU is often overexpressed in response to hypoxia, which contributes to increased risk of carcinogenesis, metastasis formation, and multiple drug resistance (MDR) development [100]. Secretory CLU has a proven ability to inhibit cell apoptosis induced by activated Bax protein or protect liver cancer cells from death by interacting with apoptogenic glucose regulated protein 78 (GRP78) [101]. Studies in which the development of HCC in rats was induced by chemical carcinogens confirmed that differences in blood plasma and hepatic CLU expression may become specific early biomarkers of hepatocyte malignant transformation [102].

Comunale et al. analyzed the glycosylation profile of serum clusterin in the context of searching for a new specific diagnostic marker for HCC [103]. Comparison of serum CLU levels in patients with liver lesions with a control group of healthy people revealed no significant differences between the analyzed groups, and the obtained results lay within the reference values range, probably due to the deliberate selection of patients with similar clusterin concentrations for the study groups [103]. The analysis of blood serum CLU glycosylation profile using DSL lectin (DSL; *Datura stramonium* lectin), specific to *β*1,4-linked N-acetylglucosamine of triantennary N-glycans, in three groups of patients (HCC, cirrhosis and control) showed a significant decrease in the expression of triantennary N-glycans in serum samples of HCC patients in comparison to controls and cirrhosis patients. In contrast, no significant differences in the glycosylation profile of CLU between patients with different stages of HCC were observed. In addition, it was noted that a reduction of expression of clusterin *β*1,4-triantennary N-glycans in HCC patients was accompanied by an increase in the expression of biantennary N-glycans [103, 104], which may have been caused by decreased expression of N-acetylglucosamine transferase IV, an enzyme which initiates the formation of an additional third branch on the biantennary glycan, resulting in the formation of triantennary glycan [105]. In conclusion, the analysis of the degree of *β*1,4-linked triantennary glycan expression in clusterin, together with the determination of AFP concentration, can be used as a screening test for patients at high risk of developing HCC as well as for monitoring the treatment of HCC patients and possible recurrence of the disease [103].

**Figure 5:**
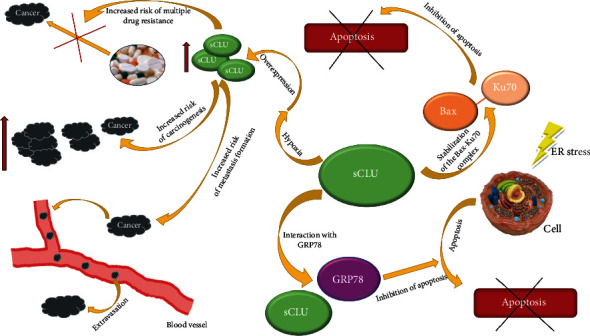
Schematic representation of the role of sCLU in the development of liver cancer. sCLU: secretory clusterin; Bax: Bcl-2-associated X protein; Ku70: Lupus Ku autoantigen p70; GRP78: glucose-regulated protein 78; ER: endoplasmic reticulum.

In summary, current literature data concerning the role of sCLU in HCC indicates that an increase of sCLU levels in the blood serum and liver may become an early marker of malignant transformation of hepatocytes and increased sCLU levels may also suggest disease progression. Another promising aspect of CLU examination in the context of liver cancer development is the analysis of its glycosylation profile. Up to date, decreased expression of triantennary N-glycans together with an increase of biantennary N-glycans in patients with HCC was revealed. Further studies in this field may not only shed new light on the molecular processes associated with hepatocellular carcinoma pathogenesis but may also contribute to the invention of some new HCC biomarkers. The role of sCLU in the development of liver cancer is schematically presented in Figure 5.

### 3.7. The Importance of the Clusterin Glycopeptide Variant in Clear Cell Renal Cell Carcinoma

Clear cell renal cell carcinoma (ccRCC) is the most common and at the same time the most aggressive renal cancer, representing 75% of cases of neoplasms of this organ. Patients with genetically determined Von Hippel-Lindau syndrome (retinal-cerebellar angioma) are particularly at risk of developing ccRCC. Clear cell renal cell carcinoma is characterized by rapid growth and metastasis formation; therefore, increasing emphasis is being placed on the identification of new markers for early detection and/or prediction of disease recurrence. Most often ccRCC is diagnosed accidentally during ultrasound or computed tomography. Detection of this cancer at an early stage enables surgical treatment to take place, while therapeutic options for advanced ccRCC are severely limited [106].

The low blood concentrations of potentially diagnostically relevant glycoproteins pose a difficult challenge for glycoproteomic studies. In addition, the observed variability of glycans attachment at many different glycosylation sites of N-glycoprotein (macroheterogeneity) and variability in the profile and degree of N-glycan expression at one or more glycosylation sites (microheterogeneity) further complicate this analysis. The most common method of N-glycosylation analysis is the determination of oligosaccharide or glycopeptide profile. Oligosaccharide profile analysis is based on the release of a single N-glycan from a purified glycoprotein, using PNGase F (peptide N-glycosidase F), which is then analyzed by chromatography or mass spectrometry. In contrast, the assessment of the glycopeptide profile is based on the analysis of a single glycoprotein or multiple glycoproteins, which are analyzed by liquid chromatography with a mass spectrometer (LC-MS) [107, 108]. The analysis of glycopeptides by LC-MS provides information on the heterogeneity of glycoprotein oligosaccharides as well as on their attachment sites, whereas the analysis of oligosaccharides provides information only on glycan structure [110].

Observation of changes associated with the glycosylation site may help to understand the mechanism of glycoprotein action and improve the specificity of glycan detection, which may have potential therapeutic applications [110–112]. Studies by Kurahashi et al. have shown that high expression of CLU in surgically removed ccRCC tumor tissue may correlate with shorter survival, even if the patient does not experience recurrence [113]. A study with the use of highly invasive Caki-1 human RCC cells with expression shRNA of clusterin, targeting clusterin (Caki-1/clusterin shRNA), documented that clusterin significantly intensifies the activity of S100A4, one of the members of the S100 proteins family occurring in human RCC, which positively affects tumor growth and invasion. CLU was also observed to enhance metastasis formation, and its expression was noticeably higher during the invasion process [62]. However, not so much is known so far about the importance of blood plasma clusterin expression in detecting or monitoring ccRCC progression. Tousi et al. analyzed blood plasma clusterin glycosylation in patients with ccRCC [114]. The authors compared the N-glycans profile of clusterin present in the blood plasma of patients before and after nephrectomy (RCC(+) and RCC(-), respectively) of subjects with diagnosed ccRCC [114]. The expressions of A2G2S(3)2 (biantennary digalactosylated disialylated glycan) and FA2G2S(3)2 (core fucosylated biantennary digalactosylated disialylated glycan) were significantly lower in patients before curative nephrectomy in comparison to the results obtained from the same patients after surgical intervention. Moreover, an increase of A3G3S(6)2 (triantennary, trigalactosylated disialylated glycan) expression was observed in the blood plasma samples of patients after surgery [114]. In subsequent studies, Gbormittah et al. [115] confirmed that the expression of FA2G2S2 (core fucosylated biantennary disialylated digalactosylated glycans) and A2G2S2 (biantennary disialylated digalactosylated glycans) best differentiate blood plasma samples of patients with ccRCC before and after nephrectomy. A significant increase in the expression of FA2G2S2 and A2G2S2 glycans was observed in RCC(-) samples, i.e., after nephrectomy of a localized tumor, whereas blood plasma samples of RCC(+) patients before nephrectomy were characterized by a significant decrease in FA2G2S2 and A2G2S(3)2 expression in clusterin glycans [115]. The results of the above studies have shown that changes in clusterin glycosylation may be used to differentiate blood plasma samples from patients with ccRCC from those without renal neoplastic changes. So far, only the total blood plasma CLU concentration of patients with ccRCC before and after nephrectomy has been determined, with mean values in the range of 285-295 *μ*g/mL, but no significant difference between these two groups of studied patients was observed. Hence, the value of total CLU concentration cannot be used as a diagnostic parameter of ccRCC development. However, glycoproteomic analysis of specific CLU glycoforms may become a useful marker for monitoring the development of ccRCC [115]. As the study performed by Gbormittah et al. [115] also documented decreased expression of core-fucosylated CLU glycans in the blood plasma of patients with ccRCC, the question arises whether ccRCC cells directly produce altered clusterin with reduced fucosylation or whether the observed changes in CLU fucosylation are a secondary effect of the influence of the tumor microenvironment [115].

Scientific studies reported that elevated clusterin expression in the ccRCC tumor tissue may correlate with shorter patient survival times. Current literature data suggests that the examination of CLU glycosylation profile in ccRCC patients is more interesting from a diagnostic point of view than the analysis of blood plasma clusterin levels. It has been demonstrated that CLU glycosylation profiles before and after nephrectomy are distinct. The expression of core fucosylated biantennary disialylated digalactosylated glycans and biantennary disialylated digalactosylated glycans in the CLU molecule significantly decreased in patients before nephrectomy in comparison to patients after nephrectomy. However, it is still an open question whether the observed changes in CLU glycosylation are the cause or the result of ccRCC development.

### 3.8. The Importance of Clusterin Glycosylation in the Pathophysiology of Neurodegenerative Diseases

Among all the human tissues examined, the highest expression of CLU is observed in brain tissue. Clusterin has been proven to play an important role in the pathogenesis of Alzheimer's disease. Elevated CLU concentrations were found in both the affected brain areas: the hippocampus and frontal cortex, as well as in blood plasma [116–118]. Based on the above findings, Desikan et al. have suggested that elevated levels of blood plasma CLU are associated with the occurrence and severity of AD as well as with increased *β*-amyloid deposition and brain atrophy; however, these changes were not observed in all AD patients [118]. The authors suggested that CLU contributes to both increased *β*-amyloid aggregation and *β*-amyloid elimination, raising the question of whether an increase in CLU concentration in AD pathogenesis is beneficial or perhaps detrimental to the patient [116–118].

Tau protein is one of the most important proteins involved in AD pathogenesis. It has been demonstrated that the concentration of this protein increases after CLU injection into the rat hippocampus [119]. Yuste-Checa et al. have proposed a cellular model describing the relationships between CLU and tau protein, pointing to the chaperone role of CLU promoting the Tau protein aggregation in the whole process [120]. Lidström et al. [121] expected that increased CLU levels in the brain tissue of AD patients would positively correlate with increased CLU levels also in the cerebrospinal fluid (CSF); however, this assumption turned out to be incorrect [121, 122]. To prove that the potential involvement of clusterin in synaptic degradation, the correlations between CLU concentrations, and the concentrations of neurogranin (NG), a potential marker reflecting synaptic degradation, were examined in CSF. A significant positive correlation was found between the concentrations of both substances in CSF, occurring independently of other factors [123]. Since clusterin is a highly glycosylated protein, Nilselid et al. [124] decided to check whether the results obtained by Lidström et al. [121] were associated with differences in the glycosylation profile of CLU derived from brain tissue and the one present in the CSF, which could disturb the binding of specific CLU-detecting antibodies, consequently leading to falsely underestimated results. Glycosylation analysis of the clusterin present in CSF showed that it contains partially sialylated N-glycans, while the presence of O-glycans was not identified. The lower molecular weight of CSF clusterin compared to blood plasma CLU was probably associated with different glycosylation degrees of the CLU present in both body fluids [124]. Pilot studies concerning determinations of native CLU concentrations in the CSF of AD patients and in control samples of healthy subjects did not show significant differences between the studied groups. The CLU deglycosylation process is also accompanied by an increased concentration of deglycosylated CLU in the CSF samples of AD patients in comparison to controls [125]. Based on the results of the above pilot study, the authors suggested the determinations of native and deglycosylated CSF CLU concentrations in larger groups of participants. Interestingly, despite the fact that deglycosylation of CLU resulted in higher deglycosylated CLU concentrations in CSF, elevated CLU concentrations of about 25% in AD patients were noted in CSF samples for both native and deglycosylated CLU. This provides evidence that the deglycosylation of CLU was not necessary to demonstrate the elevated concentrations of this glycoprotein in the CSF of AD patients, but contributed to a more efficient detection of CLU by specific antibodies when its levels were determined [125].

Sihlbom et al. [126] demonstrated the presence of clusterin glycosylation changes in the CSF of AD patients, but because these changes also affected other proteins, the researchers suspected that this was a generalized variation in glycoproteins' glycosylation in the course of AD. The elevated concentrations observed after CLU deglycosylation suggested that clusterin microheterogeneity analysis may become a severity assessment marker in AD patients and determining the profile and degree of CLU glycosylation in CSF or the total glycosylation profile of CSF glycoproteins of AD patients would enable CSF differentiation between AD patients and healthy individuals [124].

Molecular studies on the CLU gene revealed that the presence of AD risk alleles rs9331888 and rs11136000 is associated with decreased blood plasma CLU levels [127]. Moreover, the presence of the single nucleotide polymorphism (SNP) rs11136000 in the CLU gene is associated with reduced risk of late-onset Alzheimer's disease (LOAD), which is more frequent in female than in male subjects [128]. The neuroprotective role of clusterin in the pathogenesis of AD is based on its activity as *β*-amyloid transporter, thus preventing the accumulation of *β*-amyloid deposits removing them from brain tissue, simultaneously inhibiting the complement system and neuronal apoptosis process, and promoting neurite growth [129].

An ideal prognostic and diagnostic biomarker should be characterized by variability while monitoring disease progression and treatment effects, thus contributing to a more accurate and earlier diagnosis of AD. It was suggested that the altered glycosylation profile of CLU may provide important information about disease progression. As mentioned above, blood plasma and CSF clusterin contains sialylated N-glycans [15], and changes in the degree of CLU glycan sialylation are associated with AD development [130, 131]. Liang et al. reported the results of blood plasma CLU glycosylation analysis of patients with mild and severe hippocampal atrophy [132]. Three glycosylation sites, *α*64Asn, *β*64Asn, and *β*147Asn, showed significant differences in glycosylation pattern between the study groups. The greatest changes were observed in the composition of glycans attached to *β*64Asn; 8 glycoforms were identified, and their expression was significantly reduced in patients with advanced hippocampal atrophy compared to subjects in the early stages of the disease, indicating the diagnostic utility of CLU glycoform examination as a prognostic marker of AD [132].

Following the discovery of single nucleotide polymorphisms in the CLU gene that contribute to the development of Alzheimer's disease, a possible role for clusterin in the pathogenesis of other neurodegenerative diseases was suggested. Researchers have paid particular attention to Parkinson's disease (PD), which is characterized by the formation of abnormal *α*-synuclein aggregates. The expression of CLU rs9331896 allele was shown to be associated with a significantly higher risk of Parkinson's disease development in the Chinese Han population, especially in males [133]. Another study reported increased cognitive changes in PD patients homozygous for the C allele of CLU rs11136000 [134]. In a study performed on a cell line with *α*-synuclein overexpression, it was shown that reduced CLU expression enhances the formation of *α*-synuclein aggregates [135]. Clusterin levels also appear to be a potential candidate biomarker for Parkinson's disease, and blood plasma CLU levels in PD patients have been documented to be significantly higher than in healthy subjects [133]. A similar relationship was observed for CLU levels in CSF [136], but recent studies do not support these observations, reporting only increased CLU levels in CSF in healthy subjects with high-risk of PD [134].

**Figure 6:**
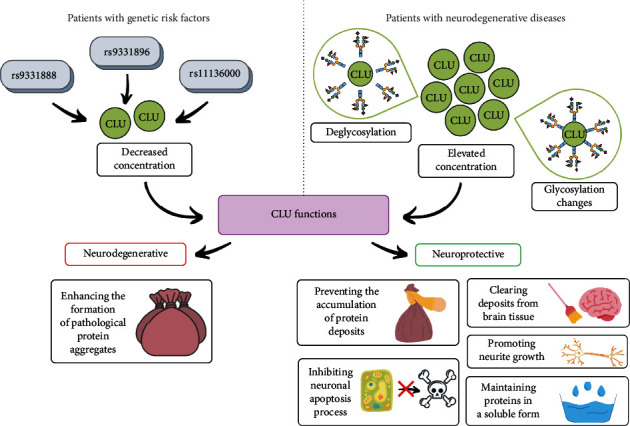
Schematic representation of the main CLU functions in neurodegenerative diseases and its possible neurodegenerative and neuroprotective activity. CLU: clusterin; rs9331888, rs9331896, and rs11136000: single nucleotide polymorphisms of clusterin gene.

In conclusion, several studies have confirmed the association between CLU and *β*-amyloid formation, indicating its role in Alzheimer's disease. Increased blood CLU levels are related to the occurrence and the severity of AD. However, the particular role of CLU in this disease, especially its role in the *β*-amyloid metabolism, is not fully understood and requires further analysis. Another aspect of this issue is the analysis of CLU glycosylation profile and degree in CSF and blood plasma. The observed changes in the profile and degree of CLU glycans sialylation in blood plasma and CSF, associated with AD development, suggest the need for further experiments concerning the analysis of CLU glycosylation heterogeneity in context of the development and progression of neurodegenerative disorders. CLU has also been documented to play an important role in Parkinson's disease, being significantly related to a higher risk of PD development, especially in males; a potential role of clusterin in preventing *α*-synuclein aggregation has also been suggested. Moreover, blood plasma clusterin levels appear to be a potential candidate biomarker for Parkinson's disease development, as they are significantly increased in PD patients in comparison to healthy subjects. The main CLU functions in development of neurodegenerative diseases as well as its neuroprotective properties are presented in Figure 6.

### 3.9. The Harmful Effects of Ethanol on Glycosylation of Clusterin Present in Brain Tissue

The destructive effect of ethanol on living organisms has been the subject of numerous studies. Most scientific reports detail the results of studies on the effects of chronic ethanol exposure on liver function [137, 138]. Long-term exposure of the body to ethanol not only results in hepatic steatosis, but can also lead to changes in liver protein synthesis and/or secretion [139].

Studies in rats have shown that ethanol selectively impairs glycoprotein metabolism, resulting in their alteration [137]. Ethanol has been documented to reduce the sialylation of brain tissue proteins [140]. The determination of the functions of glycoprotein carbohydrate structures has been the subject of studies for many years. Hale et al. analyzed how glycosylation of brain tissue proteins is affected by chronic ethanol exposure, using CLU as a model of N-glycosylated protein [71]. CLU, as one of the proteins present in brain tissue, is involved in cell aggregation, lipid transportation, remodeling of synapses, and cell membrane protection [141, 142]. The biosynthesis of CLU is regulated *inter alia* by the consistent action of two enzymes: sialotransferases and sialidases, present in brain microsomes, Golgi apparatus, cytosol, and plasma membranes [143]. Studies performed by Hale et al. [71] showed that sialylation is a key step in clusterin biosynthesis and that long-term ethanol exposure significantly impairs this stage of CLU biosynthesis. In previous studies in rats, Ghosh et al. showed that chronic ethanol exposure can lead to the modification of Golgi apparatus membranes, which form a key cell compartment for clusterin sialylation [138]. Loss of sialic acid by the clusterin molecule may result in a change in its molecular conformation, which may in turn affect its stability, antigenic expression, or receptor recognition (Figure 6). Javors and Johnson [144] proved that this process is reversible: Sialylation of CLU increased after alcohol overdose reduction. The amphipathic nature of the clusterin molecule potentially provides its ability to transport lipids, which can be incorporated into axonal membranes, maturing neurons, and neurons undergoing dendritic reorganization. In cases of nerve cell degeneration as well as mechanical, metabolic, or chemical damage of brain cells, clusterin expression can be increased due to the promotion of tissue repair and remodeling. This process probably requires the structural stability of the clusterin molecule and its correct structure [71]. Changes in degree of clusterin sialylation due to long-term ethanol exposure alter its structure, leading to impairment of its function related to the transport of lipids and neuropeptides used in ischemic brain tissue repair and remodeling [145, 146].

**Figure 7:**
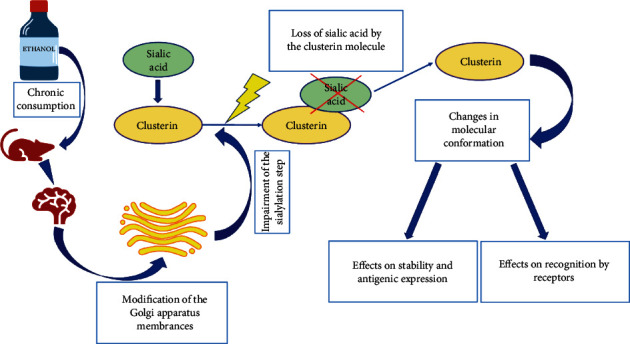
Schematic representation of chronic ethanol exposure on CLU structure and functions. Self-modification based on Ghosh et al. [138] and Hale et al. [71].

In conclusion, it has been documented that sialylation is a crucial step in CLU biosynthesis. Chronic ethanol exposure results in CLU sialylation disturbance, *inter alia* via Golgi apparatus impairment—the main compartment where the CLU glycosylation process takes place. The altered CLU structure disables its proper activity bound with lipid and neuropeptide transportation, which is crucial to ischemic brain tissue repair. The most important impacts of chronic ethanol exposure on the structure and functions of CLU are presented in Figure 7.

### 3.10. Structural Analysis of Clusterin Present in the Serum of ATTRwt Patients

Wild-type (wt) transthyretin amyloidosis (ATTR) is a disease that involves the accumulation of protein deposits in cardiac muscle fibers, causing heart failure in elderly people. These deposits result from the disassociation of the tetrameric protein transthyretin (TTR). Unlike inherited TTR amyloidosis, in which a change in amino acid sequence destabilizes the native transthyretin molecule, the amyloid protein in ATTRwt does not show an altered amino acid sequence. Therefore, scientists have searched for other factors that may be responsible for protein destabilization and the uncontrolled accumulation of wild-type TTR in myocardial fibers. The results of some research suggest that clusterin may play a role in the pathomechanism of this disease [147, 148].

Torres-Arancivia et al. [149] performed a comparative glycoproteomic analysis of CLU present in the blood sera of healthy and ATTRwt patients, which showed that the *α*81Asn clusterin glycosylation site, present in patients with this disease (CLUp), was characterized by lower diversity and glycosylation degree than in healthy patients; however, the oligosaccharide profile of glycans attached at the *β*352Asn position was similar for both CLUp and controls (CLUc). Furthermore, the glycans HexNAc:4, Hex:5, Fuc:0, and Neu5Ac:2 were frequently present at the *β*332Asn position and were identified only for CLUc. The presented structural differences between CLUc and CLUp, concerning the oligosaccharide profile, may be the reason for the reduced ability of clusterin to bind nonnative TTR, thus reducing the activity of clusterin as a chaperone and causing accumulation of wild-type TTR amyloid deposits in myocardial tissue, leading to myocardial failure [149].

In 2020, Torres-Arancivia et al. [150] published the results of a continued study on the role of CLU in the pathobiology of ATTRwt amyloidosis. They examined the amino acid content and oligosaccharide occupancy of CLU and compared the results with data obtained for control blood sera of healthy subjects. The authors concluded that there was no variation in the amino acid sequence of CLU between the sera of patients with ATTRwt and the control group, while differences between both groups were found in the degree of oligosaccharide expression in CLU. The highest amount of glycoforms in ATTRwt CLU was present at position *α*, which confirmed the hypothesis that the occurrence of alterations in glycan expression in the N-terminal region of the *α* subunit of circulating CLU may negatively affect the chaperoning capacity of clusterin in ATTRwt patients, influencing its ability to prevent the deposition of amyloid fibrils [150].

Current literature data concerning ATTRwt suggest that the differences in one of the glycosylation sites (*α*81Asn) between blood plasma CLU of ATTRwt patients and the control group of healthy subjects may lead to the reduction of clusterin ability to bind nonnative TTR and thus diminish the activity of clusterin as a chaperone, causing accumulation of wild-type TTR amyloid deposits in myocardial tissue and leading to myocardial failure.

### 3.11. The Importance of Clusterin Glycosylation in the Early Phase of Myocardial Infarction

Ischemic atherothrombotic syndromes cause pathological changes in the body, which are reflected in fluctuations of serum protein concentrations. Increased concentrations of C-reactive protein (CRP), amyloid A, or interleukin 6 (IL-6) were observed in a significant percentage in patients with acute coronary syndromes. Ischemia-modified albumin (IMA) is one of the best-known markers of myocardial ischemia [151], while the determination of troponin concentration is now a common test performed both for diagnostic purposes and for monitoring the degree of myocardial damage [152]. However, the fact that markers used to diagnose heart disease are characterized by low tissue specificity and rapid normalization of concentrations remains problematic. Moreover, in the case of the assessment of myoglobin concentration, the obtained value may be burdened with a large error among patients with renal insufficiency. From the clinicians' point of view, the time elapsed between the onset of myocardial infarction and its correct diagnosis is of crucial prognostic importance for the patient, because the immediate application of revascularization treatment of the coronary artery has the most effective results and gives the patient a chance to recover [153].

The identification of a biomarker that would indicate cardiac damage already in the first hours after an incident of myocardial infarction is still the subject of many studies. The known biomarkers used in the diagnosis of vascular atherosclerotic lesions include LDL, HDL, and apolipoproteins ApoA1 and ApoB [154, 155]. The analysis of HDL composition showed that the HDL structure includes proteins involved in the activation of the complement system, the regulation of proteolysis, and acute phase proteins [156]. In some reports, scientists suggest that the participation of HDL in the development of cardiovascular diseases is not based on its concentration in the blood but on its qualitative composition, structure, and biological function. Moreover, the results of clinical trials suggest that the HDL molecule may exert anti-inflammatory and cytoprotective effects during the ongoing inflammatory process [157, 158]. Clusterin bound to ApoA1 is a part of the HDL molecule [159], and the level of its expression in the blood serum depends on the maintenance or disturbance of the organism's homeostatic conditions [92]. The presence of high CLU concentrations in the sera of patients with diagnosed atherosclerotic lesions was observed. Cubedo et al. performed a proteomic analysis of proteins present in blood sera of patients in the early phase of acute myocardial infarction (AMI) [160]. Significant changes in the expression of individual clusterin isoforms, with a predominance of those with lower molecular weight, have been demonstrated in the sera of patients with AMI in comparison to the control group due to a reduced degree of CLU glycosylation in the sera of AMI patients. In addition, immunohistochemical analysis revealed the presence of CLU in ischemic myocardial tissue, which was not observed in myocardial muscle fibers without lesions [160]. The analysis of changes in CLU glycosylation degree may help to understand the role played by this glycoprotein in the pathomechanism of the development of AMI and may also become a more reliable biomarker to detect the early phase of AMI than parameters measured so far [160]. In addition to changes in the degree of clusterin glycosylation, a significant decrease in CLU blood serum levels was also demonstrated in patients within the first 6 hours of myocardial injury, and within 24 hours, CLU levels began to return to physiological values, reaching a value in the reference range within 72-96 hours. The observed decrease in the concentration of clusterin is probably the result of its action as an anti-inflammatory protein [160].

In summary, the presence of CLU in ischemic myocardial tissue was proven, suggesting the important role of this glycoprotein in the AMI. Moreover, serum CLU concentrations were significantly higher in the group of patients with AMI in comparison to the control group. It is worth noting that blood serum CLU levels are characterized by dynamic changes during the time after ischemia. Their levels decrease within 6 hours of ischemia and are normalized within 24 hours. The aforementioned information suggests that blood serum CLU may become an additional, sensitive myocardial infarction biomarker.

### 3.12. Analysis of Clusterin Glycoforms in the Urine of Cows with Spongiform Encephalopathy

Bovine spongiform encephalopathy (BSE) is a fatal neurodegenerative disease associated with prion infection that causes damage of the central nervous system, manifesting as vacuolization and gliosis of the grey matter of brain tissue [161]. Considering the fact that people can become infected with this disease by eating meat from sick animals, it is very important to detect it early in cattle. Formerly, the diagnosis of BSE was based on a combination of symptoms of aggressive behavior in animals and lack of motor coordination. Among deceased animals, in order to confirm the diagnostic hypothesis and unequivocally identify the cause of death, an analysis of the abnormally folded PrPd protein present in brain tissue was performed and collected post-mortem [162]. It has been shown that in transmissible spongiform encephalopathy (TSE), which includes BSE, the concentration of clusterin mRNA increases [163]. Using two-dimensional differential gel electrophoresis (2D-DIGE), Simon et al. found that only certain isoforms of clusterin showed differential expression in urine from infected cattle compared to controls [164]. The difficulty in using specific clusterin isoforms as a biomarker in BSE diagnostics is the observed background interference in the form of other proteins with identical amino acid sequences, whose presence is not characteristic for BSE [164]. Lamoureux et al. performed a study to determine the diagnostic potential of clusterin isoforms present in the urine of BSE-infected cattle [165]. The use of CAB1 and CAB2 (CAB; custom polyclonal antibodies) specific for *α* and *β* subunits of clusterin, respectively, confirmed that the detected proteins, present in the urine of BSE cattle, were isoforms of the CLU *β* chain. The 3 *β* subunits of CLU characteristic for BSE as well as 2 *β* chains with lower molecular weight and a lower isoelectric point (pI) were identified [165]. In the next step, the glycosylation profiles of three potentially diagnostically relevant clusterin isoforms, typical for BSE, were analyzed and showed reactivity with lectins specific to: hybrid N-glycan structures (WGA; *Wheat germ* agglutinin) and high mannose oligosaccharide (ConA) structures, meaning that all three clusterin isoforms have complex glycans in their structure. However, Western blot analysis showed that all urine samples from uninfected cattle were characterized by the presence of a single band, reactive with anti-CLU antibodies, at a site corresponding to a molecular weight of about 37 kDa, while urine samples from infected cattle additionally showed a second band, with a molecular weight higher than 37 kDa. Deglycosylation of CLU from the urine of affected cattle, followed by Western blot analysis, revealed the presence of a single band with a molecular weight of approximately 37 kDa, which seems to support the hypothesis that the clusterin molecule undergoes stronger glycosylation during BSE development. The authors concluded that the analysis of the degree of glycosylation of clusterin isoforms, present in a readily available biological material such as urine, could serve as a basis for the creation of a quick and specific diagnostic test that could be applied to the diagnosis of BSE in breeding cattle [165].

To summarize, studies concerning the usefulness of CLU as a potential biomarker of bovine spongiform encephalopathy showed differential expression of CLU in the urine of infected cattle in comparison to the healthy controls. Glycosylation profile analysis revealed that CLU in BSE had a stronger degree of glycosylation as an effect of disease occurrence and progression. Further analysis of urine CLU glycosylation may result in the development of a quick and specific diagnostic test. Such a test could be applied to the diagnosis of BSE in breeding cattle in an easy and convenient way, especially in the context of preventing the transmission of the disease from infected cattle to humans.

Table 1 summarizes the main changes in expression of sCLU glycans in selected disorders.

## 4. Conclusions

Clusterin is a very interesting glycoprotein with a multifunctional role in the human body. Several pieces of research have proven that its concentration as well as its structure differs in many pathological conditions. The glycosylation process is essential for proper CLU biosynthesis, enabling chaperone activity as well as the formation of soluble complexes with misfolded proteins. In such a manner, damaged proteins are then removed by endocytosis and lysosomal degradation in an ATP-independent way, thus playing a great role in homeostasis maintenance. With its proapoptotic properties, the nuclear form of clusterin present in neoplastic cells inhibits tumor progression, whereas secretory clusterin performs chaperone activity and may contribute to metastasis formation. It is worth pointing out that CLU lacking in N-glycans is cumulated in the ER, which results in cytotoxicity induction. The presence of oligosaccharide structures type Le^x^/Le^a^ and Le^y^/Le^b^ enable CLU and DC-SIGN interactions, which influence the immunological properties of seminal plasma as well as neoplastic cell modulation and induce macrophage differentiation, leading to proangiogenic cell formation. Since CLU plays a variety of important roles in the human body, further studies concerning the use of CLU as a potential biomarker in many civilizational diseases may shed more light on the molecular mechanisms involved in the pathogenesis of these disorders and, in addition, may also contribute to the development of new therapeutic strategies.

## Figures and Tables

**Table 1:** The main changes in expression of sCLU glycans in selected disorders.

Disorder	Examined material	CLU glycan expression	Reference
Human reproduction	Blood serum	(i) Increased expressionof core and *α*1,2-linked fucosein infertile normozoospermic patientsin comparison to male patientswith sperm disorders	[33]
		(ii) Possibility of different CLU glycosylationmechanismsin seminal plasma and serum CLU	
	Semen, human oocytes	(i) Expression of Le^x^ and Le^y^ oligosaccharide structures in seminal CLU make possible the reaction with DC-SIGN expressed in DCs of zona pellucidaand induction of maternal immunotolerance to male antigens	[32, 33, 78]

Breast cancer	Tumor tissue	(i) Ability to metastases formation through neoexpression of sialo-Le^x^ structures	[86, 87]
		(ii) Increased expression of fucose of Le^x^ and Le^y^ structures in tumor tissues in comparison to healthy nontumor tissue from the same patient	[88]
		(iii) Ability of fucosylated CLU, present in neoplastic cells, to bind to DC-SIGN	
		(iv) Fucosylated form of CLU produced by tumor cells as a possible breast cancer advancement marker	

Colorectal cancer	Blood serum	(i) Increased expression of five CLU isoforms in group of patients with CRC in the fraction containing O-glycosylated glycoproteins, but without N-glycans	[92]
		(ii) Increased expression of one CLU isoform in group of patients with CRC in fraction of N-glycosylated glycoproteins	
	Tumor sections	(i) Expression of highly glycosylated cytoplasmic CLU in the advanced stage of cancer with metastases to the lymph nodes	[90]
		(ii) Extracellular expression of highly glycosylated CLU in the advanced CRC	

Hepatocellular carcinoma	Blood serum	(i) Significant decrease in the expression of triantennary N-glycans in serum samples of HCC patients in comparison to controls and cirrhosis patients	[103]
		(ii) Reduction of expression of *β*1,4-triantennary N-glycans of clusterin in HCC patients, accompanied by an increase in the expression of biantennary N-glycans	

Clear cell renal cell carcinoma	Blood plasma of patients before and after nephrectomy	(i) Significant decrease in the levels of a biantennary digalactosylated, disialylated glycans (A2G2S2)	[114]
		(ii) Increased levels of a core fucosylated biantennary digalactosylated, disialylated glycans (FA2G2S2)	
		(iii) Increase of triantennary trigalactosylated, disialylated glycans (A3G3S(6)2) in blood plasma post-surgery	
		(i) Increased expression of FA2G2S2 (core fucosylated biantennary digalactosylated, disialylated glycans) and A2G2S2 (biantennary digalactosylated, disialylated glycans) in blood plasma of patients following nephrectomy	[115]
		(ii) Expression of FA2G2S2 (core fucosylated biantennary digalactosylated, disialylated glycans) and A2G2S2 (biantennary digalactosylated, disialylated glycans) as a differentiating biomarker of patients with ccRCC before and after nephrectomy	

Alzheimer's disease	Cerebrospinal fluid	(i) CLU glycans partially sialylated	[124]
		(ii) Lack of O-glycans in the CSF CLU molecule	[125]
		(iii) Elevated deglycosylated CLU concentrations in the CSF samples of AD patients compared to controls	
		(iv) Decreased CLU sialylation degree	[130, 131]
	Blood plasma	(i) Decreased expression of the eight glycoforms attached to the *β*64Asn site in patients with advanced hippocampal atrophy compared to those in the early stages of the disease	[132]

Chronic ethanol overdose	Rat brain tissue	(i) Sialylation as a key step of the CLU biosynthesis	[71]
		(ii) Desialylated form of CLU as a result of chronic ethanol overdose	
	Blood serum	(i) Decreased blood serum CLU sialylation in alcohol-overdosing patients	[144]
		(ii) Reversibility of CLU desialylation process during abstinence	

Wild-type (wt) transthyretin amyloidosis	Blood serum	(i) Lower diversity and glycosylation degree of CLU glycans attached to *α*81Asn glycosylation site in comparison to the control group	[149]
		(ii) Similar oligosaccharide profile of glycans attached at the *β*352Asn position	
		(iii) CLU glycan sequence characteristic for patients with ATTRwt: HexNAc:4, Hex:5, Fuc:0, and Neu5Ac:2 frequently present at the *β*332Asn position	
		(i) No variation in the amino acid sequence of blood serum CLU between the group of patients with ATTRwt and the control group	[150]
		(ii) Differences in the degree of oligosaccharide expression in CLU between the ATTRwt patients and control group	
		(iii) The highest amount of CLU glycoforms in ATTRwt, present at position *α*—confirmation of the hypothesis that the alterations in glycan expression in the N-terminal part of the *α* subunit of circulating CLU may negatively affect the chaperoning capacity of clusterin in ATTRwt patients, influencing its ability to prevent the deposition of amyloid fibrils	

Acute myocardial infarction	Blood serum	(i) Reduced CLU glycosylation degree in group of AMI patients in comparison to the healthy control group	[160]

Bovine spongiform encephalopathy	Cattle urine	(i) Differential expression of certain isoforms of clusterin in urine of infected cattle compared to controls	[164]
	Cow urine	(i) Identification of high mannose complex CLU N-glycans	[74]
		(ii) Different CLU isoform expression in infected and uninfected cow urine	

AD: Alzheimer's disease; AMI: acute myocardial infarction; Asn: asparagine; ATTRwt: wild-type (wt) transthyretin amyloidosis; CRC: colorectal cancer; CSF: cerebrospinal fluid; DC-SIGN: dendritic cell–specific intercellular adhesion molecule-3-grabbing non-integrin; Fuc: fucose; HCC: hepatocellular carcinoma; Hex: hexose; Le^x^: Lewis^x^ oligosaccharide structure; Le^y^: Lewis^y^ oligosaccharide structure; Neu5Ac: N-acetylneuraminic acid.

## Abbreviations

2D-DIGE: Two-dimensional differential gel electrophoresis
2D-PAGE: Two-dimensional gel electrophoresis
A2G2S(3)2: Biantennary disialylated digalactosylated glycan
AD: Alzheimer's disease
AFP: *α*-fetoprotein
AMI: Acute myocardial infarction
ApoA1: Apolipoprotein A1
ApoB: Apolipoprotein B
ApoJ: Apolipoprotein J
AT: Asthenoteratozoospermia
ATP: Adenosine triphosphate
ATTRwt: Wild-type transthyretin amyloidosis
Bax: Bcl-2-associated X protein
BSE: Bovine spongiform encephalopathy
CAB: Custom polyclonal antibodies
ccRCC: Clear cell renal cell carcinoma
CLI: Complement-lysis inhibitor
CLU: Clusterin
CLUc: Clusterin concentration in control group
CLUp: Clusterin concentration in patients
ConA: *Concanavalin A* agglutinin
CRC: Human colorectal cancer
CRP: C-reactive protein
CSF: Cerebrospinal fluid
DCs: Dendritic cells
DC-SIGN: Dendritic cell–specific Intercellular adhesion molecule-3-grabbing non-integrin
DNA: Deoxyribonucleic acid
DSL: *Datura stramonium* lectin
DTT: Dithiothreitol
ECM: Extracellular matrix
ELISA: Enzyme-linked immunosorbent assay
EMT: Epithelial-mesenchymal transition
ER: Endoplasmic reticulum
FA2G2S(3)2: Core fucosylated biantennary disialylated digalactosylated glycan
GRP78: Glucose-related protein 78
HBV: *Hepatitis B* virus
HCC: Hepatocellular carcinoma
HCV: *Hepatitis C* virus
HDL: High density lipoprotein
iCLU: Intracellular clusterin
IL-6: Interleukin 6
IL-8: Interleukin 8
IMA: Ischemia-modified albumin
Le^x^: Lewis^x^ oligosaccharide structure
Le^y^: Lewis^y^ oligosaccharide structure
LC-MS: Liquid chromatography with mass spectrometry
LDL: Low density lipoprotein
LRP2: Low density lipoprotein-related protein 2
MAC: Membrane attack complex
MCF-7: Michigan Cancer Foundation 7 cells
M-CSF: Macrophage colony-stimulating factor
MDR: Multiple drug resistance
mRNA: Messenger ribonucleic acid
nCLU: Nuclear clusterin
N: Normozoospermia
Neu5Ac: N-acetylneuraminic acid
NG: Neurogranin
NK: Natural killers
OAT: Oligoasthenoteratozoospermia
OS: Oxidative stress
PD: Parkinson's disease
PGPF: Plasmin-generated protein fragments
PKB: Protein kinase B
PNGase F: Peptide N-glycosidase F
RCC(-): Patients after nephrectomy
RCC(+): Patients before nephrectomy
ROS: Reactive oxygen species
SA: Sialic acid
sCLU: Secretory clusterin
SNP: Single nucleotide polymorphism
SP-40,40: Secretory protein-40
T: Teratozoospermia
Tg: Thapsigargin
TGF-*β*: Transforming growth factor *β*
Tm: Tunicamycin
TNF-*α*: Tumor necrosis factor *α*
TNM: Tumor node metastasis scale
TSE: Transmissible spongiform encephalopathy
TTR: Transthyretin
UPR: Unfolded protein response
VEGF: Vascular endothelial growth factor
VLDL: Very low density lipoprotein
WGA: *Wheat germ* agglutinin
ZP: Zona pellucida.
